# Pathways to care with HIV-associated cryptococcal meningitis in Botswana and Uganda: Findings from a qualitative methods study

**DOI:** 10.1016/j.ssmqr.2023.100350

**Published:** 2023-12

**Authors:** David S. Lawrence, Agnes Ssali, Neo Moshashane, Georgina Nabaggala, Lebogang Maphane, Thomas S. Harrison, David Meya, Joseph N. Jarvis, Janet Seeley

**Affiliations:** a Department of Clinical Research, Faculty of Infectious and Tropical Diseases, London School of Hygiene and Tropical Medicine, UK; b Botswana Harvard AIDS Institute Partnership, Gaborone, Botswana; c Social Aspects of Health Programme, MRC/UVRI & LSHTM Uganda Research Institute, Entebbe, Uganda; d Department of Global Health and Development, Faculty of Public Health and Policy, London School of Hygiene and Tropical Medicine, UK; e Institute of Infection and Immunity, St George's University London, London, UK; f Clinical Academic Group in Infection and Immunity, St George's University Hospitals NHS Foundation Trust, London, UK; g MRC Centre for Medical Mycology, University of Exeter, Exeter, UK; h Infectious Diseases Institute, Makerere University, Kampala, Uganda

**Keywords:** HIV, Advanced HIV disease, Cryptococcal meningitis, Qualitative research, Differentiated service delivery

## Abstract

HIV-associated cryptococcal meningitis remains a key driver of AIDS-related mortality. Mortality is twice as high in those who present later to care and with severe symptoms such as confusion. We embedded a qualitative methods study within a randomised controlled trial in Gaborone, Botswana and Kampala, Uganda with the aim of understanding pathways to care. We conducted in-depth interviews with trial participants and surrogate decision makers and analysed data thematically. Between January 2020 and June 2021 we interviewed 58 individuals. Pathways to care were prolonged because headaches were disregarded by participants and healthcare workers as a common occurrence with a broad differential diagnosis of predominantly benign aetiologies. There was also a lack of awareness of cryptococcal meningitis, and it was often after HIV was diagnosed or disclosed that the pathway accelerated, resulting in hospital admission. We outline key recommendations to reduce mortality and argue for the integration of social and behavioural interventions within differentiated service delivery models for advanced HIV disease.

## Highlights

• HIV-associated cryptococcal meningitis causes 19% of all AIDS-related mortality.
• Qualitative methods study exploring pathways to care in Botswana and Uganda.
• Pathways to care were long and convoluted, resulting in worsening of the condition.
• The primary symptom, headache, was attributed to common, benign aetiologies.
• There was a lack of awareness of the condition among patients and providers.

## 1 Introduction

An estimated 650,000 people died from AIDS-related complications in 2021 (UNAIDS, 2022). This figure is a 68% reduction from the peak of 2.1 million people who died in 2004 (UNAIDS, 2004), however over the last decade the rate of decline has decreased significantly. In 2020 UNAIDS set a target to reduce annual AIDS deaths to below 250,000 by 2025 (UNAIDS, 2020) but if current trends continue 460,000 people are projected to die of AIDS-related causes in that year. These deaths occur primarily in individuals with advanced HIV disease (AHD) who have a CD4 count of less than 200 cells/μL and are vulnerable to potentially fatal opportunistic infections such as tuberculosis and cryptococcal meningitis, and malignancies such as lymphoma (Egger et al., 2002).

There remains a relatively constant burden of advanced HIV disease among people living with HIV (Carmona et al., 2018). This is an extremely heterogeneous population but can be crudely categorised into two groups. The first are individuals who have AHD upon initial diagnosis of HIV, indicating that a considerable length of time has lapsed between acquiring HIV and undergoing testing. Recent data from South Africa (Carmona et al., 2018), Nigeria (Otubu et al., 2022), and Botswana (Leeme et al., 2021) indicate that roughly 32.9%, 47.6% and 24.8% of people have AHD at diagnosis. The second group are individuals who have been diagnosed with HIV and develop AHD over time. This may be because of a number of factors including imperfect linkage to care; ART toxicity and intolerance; difficulties with adherence; and drug resistance (Ehrenkranz et al., 2021). Data suggest that this is an increasingly large proportion of people with AHD and that it is not uncommon for individuals to move ‘backwards’ along the care cascade and develop AHD in the process. For example, data from Botswana found that between 2015-16, 40% of all individuals with a CD4 count <100 cells/μL were new to care compared to 26% in 2018–19 (Lawrence et al., 2021a).

HIV-associated cryptococcal meningitis is the second leading cause of AIDS-related mortality and is estimated to cause 19% of all AIDS-deaths (Rajasingham et al., 2022). As with AHD, the burden of cryptococcal meningitis persists. Recent programmatic data from South Africa and Botswana indicate that the number of cases has stayed relatively constant in recent years (Osler et al., 2018; Tenforde et al., 2017). Cryptococcal meningitis primarily affects people with very advanced HIV disease, typically with a CD4 count less than 100 cells/μL (Lawrence et al., 2019). Meningitis is the most serious manifestation of cryptococcal disease, which is caused by *Cryptococcus* spp*,* a ubiquitous fungus that enters the lungs through inhalation of spores. In immunocompetent individuals this exposure rarely leads to any disease or impact on health, however among individuals with severely weakened immune systems, such as those with advanced HIV disease, the fungus can spread throughout the body, including to the brain. This spread is a state called cryptococcal antigenaemia and can be detected by a point of care blood test called a cryptococcal antigen (Jarvis et al., 2009). Screening the blood of people with advanced HIV provides the opportunity to identify the presence of *Cryptococcus* in the blood to attempt to avert its onward spread, and many high-prevalence countries have national screening programmes (Greene et al., 2021). If meningitis does occur the prevailing symptom is headache, and this can be followed by a myriad of other symptoms including confusion, seizures, and coma. Left untreated, cryptococcal meningitis is uniformly fatal. Death can arise from the direct impact of the fungus on the brain but also from impedance of the normal flow of fluid around the brain which leads to raised intracranial pressure and can result in coning, whereby the brainstem is pushed down through the base of the skull (Lawrence et al., 2019). Cryptococcal meningitis must be diagnosed with a lumbar puncture whereby a needle is inserted into the bottom of the spinal column to obtain cerebrospinal fluid and the same procedure is also warranted, often daily, to reduce raised intracranial pressure (Lawrence et al., 2019).

Outcomes among individuals diagnosed with cryptococcal meningitis have historically been very poor with roughly 70% of patients dying within a year (Gaskell et al., 2014; Longley et al., 2008; Nussbaum et al., 2010; Rothe et al., 2013). There have been significant advances in recent years following two landmark trials which have demonstrated that mortality rates below 30% are possible. The ACTA trial ultimately led to the World Health Organisation (WHO) in 2018 adopting a treatment regimen of a week of intravenous amphotericin B deoxycholate given with oral flucytosine as their recommended first-line treatment regimen (Molloy et al., 2018). Observational data from South Africa found the mortality gains in the trial to also be possible in routine care settings (Mashau et al., 2022). Following this the AMBITION-cm trial found a single, high dose liposomal amphotericin-based regimen to be non-inferior to the ACTA regimen (Jarvis et al., 2022) and, due to the added convenience of a single intravenous regimen, this was adopted by WHO as the first-line regimen in 2022 (World Health Organisation, 2022).

Despite the improved outcomes observed in recent clinical studies, the case fatality rate is still high compared to other opportunistic infections (Mabunda et al., 2014) and the epidemiological data suggest that cryptococcal meningitis will remain a significant contributor to mortality in the coming years (Rajasingham et al., 2022). To date there has been very limited information on the pathways to care of those individuals diagnosed with cryptococcal meningitis, primarily because of the severity of the infection and the poor outcomes (Link et al., 2022). Qualitative methods research can provide valuable insights into the lived experience of individuals diagnosed with cryptococcal meningitis that could be used to improve care and outcomes across the HIV care continuum. First, these individuals have already had HIV for a number of years and have either not been tested and/or been maintained on effective ART. Exploring and learning from their experience of living with HIV and developing AHD can inform approaches to care that stretch far beyond cryptococcal meningitis. Second, in the case of cryptococcal antigenaemia there is a window of opportunity for healthcare systems to intervene and prevent meningitis which may not always be realised, and qualitative research could help highlight areas for improvement in healthcare delivery. Third, cryptococcal meningitis typically causes what begins as a mild headache that worsens over days and weeks before leading to more severe symptoms such as confusion, seizures and coma. Mortality rates are more than double in those with severe symptoms suggestive of delayed presentation (Jarvis et al., 2022) and qualitative methods research can explore whether individuals are aware of cryptococcal meningitis and the need to present to care soon after symptoms develop. We conducted a qualitative methods study with patients diagnosed with cryptococcal meningitis and their caregivers to begin to understand their pathways to care and identify recommendations to avert mortality.

## 2 Methods

We embedded an ethnographic study entitled The Lived Experience Of Participants in an African RandomiseD trial (LEOPARD) within the AMBITION-cm trial at the Gaborone, Botswana and Kampala, Uganda sites (Lawrence et al., 2021b). In Botswana the participants were recruited at Princess Marina Hospital and in Kampala at Kiruddu Referral Hospital. AMBITION-cm is described in more detail elsewhere (Jarvis et al., 2022) and was a non-inferiority phase-III trial of a single, high-dose of liposomal amphotericin given with 14 days of flucytosine and fluconazole in comparison to the WHO defined standard of care: 7 days of amphotericin B given with 7 days of flucytosine and followed by 7 days of fluconazole. AMBITION-cm recruited 844 participants from eight hospitals in five countries: Botswana, Malawi, South Africa, Uganda, and Zimbabwe. The AMBITION regimen was found to be non-inferior in terms of averting all-cause mortality and was also associated with significantly fewer adverse events. It has since been recommended as the first-line treatment regimen for cryptococcal meningitis by the World Health Organisation (World Health Organisation, 2022).

We conducted in-depth interviews (IDIs) and direct observations, collecting data from three categories of individuals: trial participants, surrogate decision makers (SDMs) who provided consent for the trial in cases where potential participants lacked decision making capacity, and researchers working on the trial. This paper draws on data from trial participants and SDMs only. Pathways to care with cryptococcal meningitis was one of the core areas of enquiry for the LEOPARD study.

Consecutively eligible trial participants were approached to participate in two in-depth interviews. We aimed to recruit a maximum of 20 participants from each site (Kampala and Gaborone), 40 in total. We included individuals who upon entry into the trial were deemed to have decision making capacity (i.e., decision orientated) and those who were not (i.e., decision disorientated). We anticipated 30% of all trial participants to be decision disorientated at baseline but aimed for this group to make up half of all participants in this qualitative methods study. At the time of enrolment into LEOPARD all individuals must however have regained decision making capacity to consent for the IDI. In line with the epidemiology of cryptococcal meningitis we aimed for 50–60% of participants to be male (Lawrence et al., 2021c). The first IDI took place at least six weeks into the ten-week trial and the other at least four weeks after the final trial appointment. Secondly, consecutively eligible surrogate decision makers were approached to participate in a single in-depth interview at least six weeks after having provided consent for a trial participant who was decision-disorientated at baseline. We aimed to recruit a maximum of 15 individuals from each site, 30 in total, with no specification for gender. Additionally, we conducted direct observations of the research process, including the informed consent process and the administration of study drugs. The direct observations occurred after they had reached hospital so were used to contextualise the severity of the illness rather than contributing significantly to an analysis of pathways to care.

Interviews followed a topic guide tailored to each group of participants and were conducted in Setswana or English in Botswana and Luganda in Uganda. The topic guides explored the experience of developing cryptococcal meningitis (or caring for someone who had), being approached and deciding to enrol in the trial, and the experience whilst in the trial. All interviews were audio-recorded. Interviews were transcribed and translated, and field notes were made. These data were then entered into NVivo 12 and analysed using thematic analysis (Braun & Clarke, 2006). Thematic analysis involved six steps: familiarisation with data, initial code generation, searching for themes, reviewing themes, defining and naming themes and presenting final conclusions. In addition, we extracted data from the participant IDIs and summarised their pathways to care with a focus on their HIV and ART history, how long they had been symptomatic with cryptococcal meningitis, and the various interactions they had with healthcare services whilst symptomatic and prior to their admission to the AMBITION-cm trial hospital.

The research group was led by DSL who is an HIV clinician and was the lead clinician for the AMBITION-cm trial, providing direct clinical care to trial participants in Gaborone, Botswana and overseeing the clinical management of all participants recruited into the trial which included regular site visits. DSL conducted direct observations which were documented in extensive fieldnotes and reflections throughout the research process, complementing the data collected during the interviews (Lawrence, 2023). GN conducted IDIs in Uganda under the supervision of AS and JS, all of whom were independent of the trial. NM was also independent of the trial and conducted IDIs in Botswana under the supervision of DSL and with administrative support from LM. The research team were all experienced in qualitative data collection, received protocol specific training, and met regularly to discuss the content of interviews and reflect on the data.

The study was approved by the Human Resource Development Council, Gaborone (HPDME: 13/18/1); Makerere School of Health Sciences Institutional Review Board, Kampala (REF: 2019-061), Uganda National Council for Science and Technology (REF: SS386ES) and the London School of Hygiene and Tropical Medicine (REF: 17957). All participants signed a written consent form which was approved by the relevant research ethics committees.

## 3 Results

Between January 2020 and June 2021, we recruited a total of 58 individuals. Thirty-eight trial participants (18 in Gaborone, 20 in Kampala) and twenty SDMs (9 in Gaborone, 11 in Kampala). Of the 38 trial participants who took part in an IDI, 17 (45%) were female, and half were decision-disorientated at baseline. 20 were Ugandan, 12 Motswana, and six Zimbabwean. All but one (97%) presented with a headache with a median duration of 14 days (range 3–90 days), consistent with the overall trial where 96% of all participants presented with a headache of median duration 14 days. Twenty-two participants (58%) had a previous HIV diagnosis and 16 (42%) were newly diagnosed with HIV when they were admitted with cryptococcal meningitis, compared to the main trial where 30% of participants were newly diagnosed with HIV. Of the 22 with a known HIV diagnosis, 19 had previously received ART and 3 had never started. Among those 19 on ART, 8 (42%) were reportedly adherent and/or had a suppressed viral load; 6 (32%) stated their adherence was poor and 5 (26%) had defaulted and stopped taking ART entirely. When tabulating the number of healthcare interactions from the onset of symptoms to admission to the AMBITION-cm recruiting hospital the median number was 2 visits (range 0–8 visits). For those with a new diagnosis of HIV, the median number of healthcare interactions was 3 visits (range 0–8 visits) and for those with a known HIV diagnosis the median number of healthcare interactions was 2 visits (range 0–5 visits).

Within our analysis we will discuss the significance of a headache being an often-innocuous symptom and the resultant delays in recognising that a headache could be a manifestation of a serious infection; the importance in identifying that the headache was occurring in the context of AHD; and the missed opportunities to engage in HIV care throughout the illness.

### 3.1 Suspecting the headache is serious

*‘Just a simple headache, an everyday one’*

Male participant, 44 years old, newly diagnosed with HIV, Gaborone.

One of the challenges in recognising the life-threatening diagnosis of cryptococcal meningitis was that, for many participants, headaches were a common and everyday phenomenon. The headaches were often described as starting off as quite mild, and potentially being attributed to dehydration, the weather, or stresses in life such as relationship difficulties and money worries.

*‘I used to get temperatures of some sort and I would get on and off headaches and I was advised to drink a lot of water and when I used to take it the condition would improve a bit. On waking up I would feel the neck paining on one side and I would not be able to turn the neck well. I used to think that I had slept heavily on one side and I thought that was the cause.’*

Male participant, 44 years old, previously diagnosed with HIV, Kampala.

As a result, they were often managed by drinking plenty of water or taking simple analgesia kept in the house or sourced from local pharmacies and clinics, or herbal preparations that were rubbed on the head.

*‘For two weeks the headache bother[ed] me … I used to cry and talk a lot and my mother tried to reduce the pain of the headache by mixing pumpkin leaves and together with the leaves of the plant we use to sweep the courtyard and pour the mixture on my head, but the headache persisted.’*

Female participant, 23 years old, previously diagnosed with HIV, Kampala.

The headaches would initially respond to these, at least during the day, and then frequently became worse at night when the participants were lying down.

*‘I felt a very severe headache. It was the first time I came across it. I stayed something like three or four days not going to the hospital because it would subside because it would only ache at night. In the afternoon it would be quiet. Every morning it would be quieter then I would think it has healed. Then when it was time for me to go to bed it would start.’*

Male participant, 37 years old, new diagnosis of HIV, Gaborone.

For some participants this relatively indolent presentation could go on for weeks and weeks, becoming more irritating but not always much more severe or signalling a serious underlying pathology. Some participants described going back and forth to the same clinics every week or two for a healthcare worker to review their symptoms and prescribe them more, or stronger, analgesia and this was true of both those who were aware and unaware of their HIV status. In these cases, it was only when the symptoms evolved and, for example, they developed double vision, had seizures, or collapsed, that they were prompted to seek healthcare from larger health centres or hospitals.

*‘[I had spent] almost three months going to the hospital, going to the hospital, going to the hospital. They were saying if it does not get better come back again. I was going and they will say come back again. So I kept on going, maybe after 2 weeks, after 2 weeks, after 2 weeks. Even going to different hospitals.’*

Female participant, 24 years old, newly diagnosed with HIV, Gaborone.

When considering pathological causes of the headache participants developed their own differential diagnosis which was often broad such as flu, malaria which is common in Kampala but not in Gaborone, and later in the study, Covid-19. These were pathologies they had regularly encountered and could also explain the fevers which commonly accompanied the headache. This self-diagnosis could be managed by visiting pharmacies which sell antimalarials and a variety of flu remedies without the need for a clinic consultation or prescription which would help save time and money. One of the difficulties in being able to recognise meningitis was that almost no participants had ever heard of it before. Some of those who had heard of meningitis had not identified it as something they were at risk of developing with one 32-year-old female participant newly diagnosed with HIV in Kampala telling us that *‘I am an adult, not a child. I hear that children are the ones who suffer from meningitis’*. Only one 49-year-old male participant newly diagnosed with HIV in Kampala had suspected he had meningitis, having been hospitalised many years ago for another reason and seeing a case on the same ward who *‘was all straight and stiff as a dead body, so whenever my neck became tight, my thoughts went to that man’* and this prompted him to seek urgent medical attention.

In both locations, but more so in Kampala, we also found a small number of examples whereby the headache was attributed to witchcraft and having been bewitched. This was most commonly in cases whereby the participant's behaviour had changed, perhaps as a result of confusion or hallucinations. In one scenario, a 35-year-old male participant with a previously known HIV diagnosis in Kampala had fallen ill during a trip to his ancestral clan shrine and his symptoms were misinterpreted as possession and he was severely beaten with a stick by his relatives. The belief that witchcraft was the cause prompted the use of traditional medicine, either obtained from the house or from a traditional healer and in the scenario described above the traditional healer recognised that the presentation was likely related to advanced HIV disease and diverted the participant to a hospital. We also commonly encountered individuals who did not express any concerns about witchcraft but did use a combination of biomedical and traditional medicines to try and alleviate their symptoms, as they would typically for other symptoms, and sometimes visited a traditional healer after several unsuccessful trips to a biomedical facility.

### 3.2 Suspecting the headache is related to HIV

*“They sent me away from the health facility. They even took me to [a psychiatric hospital], thinking that maybe I had run mad. So, I stayed [there]. There were some who used to … wonder and ask me, saying, “You seem not to be a mad patient like others.”*

Female participant, 46 years old, previously known HIV diagnosis, Kampala.

The majority of participants had never heard of meningitis and therefore this was not commonly considered as a potential diagnosis. In some of these scenarios the HIV status was undiagnosed and therefore unknown to all, in some the diagnosis was known only by the participant, and in others all parties were aware. Those who did not know their HIV status were also often for the first-time experiencing symptoms of untreated infection including weight loss and skin changes. Some wondered if these changes and their headache could be related to undiagnosed HIV infection, and this was sometimes combined with suspicion or knowledge that their partner was also living with HIV. In these scenarios several participants used this as a prompt to go and test and this new diagnosis often triggered consideration of HIV-related pathology by clinicians. Those who knew their HIV status but were aware that they were either not on treatment at all or had been taking it infrequently did not commonly express to our team that they had considered their headache and associated symptoms to be related to HIV, although this might have been the case. In addition, nobody said that they had been told by a healthcare worker that a headache could be a serious consequence of untreated HIV. In several of these scenarios, the participants did not disclose their HIV status to healthcare workers when visiting facilities with their headache and were often tested further down the pathway, for example when they were finally admitted to hospital. Others went and tested at clinics, seeking confirmation of their diagnosis, but indicating that it was their first time to test. Finally, those who were on treatment did not say that they had been told a headache could be a serious complication of HIV. Those who had recently been started on treatment also did not indicate that they had been told that a headache could emerge shortly after starting treatment and that this could be a potentially fatal complication.

Most of our participants (all except three) had encounters with healthcare workers during the course of their symptoms and prior to reaching hospital. In one case there had been at least eight separate attendances. Quite often a potential cause for the headache was not offered by healthcare workers and in others there were a number of alternative diagnoses considered, including one 23-year-old female participant with previously known HIV diagnosis in Kampala who was told she had *‘on and off malaria’*, multiple male participants in Gaborone who were given *‘migraine pills’*, and a 34-year-old female participant in Kampala who was told she could not have HIV because she was *‘not very small’*. This lack of a diagnosis led to some participants enduring long, convoluted pathways, navigating multiple healthcare facilities, medical specialities, cadres of healthcare worker, and excessive out-of-pocket expenses whilst their symptoms continued, worsened and evolved. Several people were sent to psychiatric hospitals, some for outpatient assessments and others were admitted, including the participant quoted above. Here we present the pathway to care for one 45-year-old male participant with a previously known HIV diagnosis in Kampala.

*‘He was working away from home and developed fevers and a headache. Thinking he had developed malaria he went to a clinic in Kitende for some treatment. He took the treatment and carried on working but the headache persisted. He then started his journey back home but stopped mid-way at Salama and went to another clinic where he was diagnosed with typhoid and given some intravenous treatment for a day. He went home to Masajja and started a new job for two days but started feeling even weaker so went to another clinic, thinking that perhaps it was a very severe case of malaria. At that next clinic in Masajja he was diagnosed with brucellosis and given a dose of intravenous treatment. He went back to work but then became confused and lost consciousness. He recovered to an extent but the next day he slept all day and was taken to the same clinic in the evening for another dose of treatment for brucellosis, and again the day after. His family came to see him and took him to a clinic in Salama but they had a problem in the lab and could not do any tests, so he went to another clinic and was given some more intravenous treatment before going home. That night he struggled to sleep, and the pain became more severe: he pulled out his intravenous line, fell down and his eyes rolled to the back of his head. The family resolved to take him to the hospital the next day. That next day they looked for a suitable facility in Salama but failed to get one and ended up at a hospital in Bunga where he was admitted for 36 hours. It was as he became more unwell that he was transferred to Kiruddu. At this point he had a severe headache and intermittent confusion. He describes this entire process as taking a month. He was diagnosed with HIV in 2005 by community testing services. Initially he doubted the result. Two years later he went to another facility and tested positive again. He never started treatment and was tested again at Kiruddu. He did not mention the HIV status to anyone during this entire process until he went to Kiruddu.’*

It was common to hear that patients moved between these multiple facilities whilst their health deteriorated and it was often after they developed symptoms of severe infection, such as confusion, collapse, seizures, or coma that the diagnosis of meningitis was considered. What was clear however was that upon recognition of advanced HIV disease and meningitis the pathway moved much faster, and participants described that they were rushed to hospitals, with the AMBITION-cm recruitment hospitals being clearly recognised as the appropriate facility for patients to be transferred. In Kampala in particular, it was very clear that Kiruddu Hospital was the specialist centre to manage meningitis and all other hospitals referred participants here. In Gaborone it was typically the case that participants were sent to Princess Marina Hospital but there were some scenarios whereby they first presented to private hospitals in extremis but after the diagnosis had been made, they were informed about the likely length and cost of the hospital admission and instead had to transfer to the government facility.

### 3.3 Missed opportunities in HIV care

We identified several ways that HIV care had missed the opportunity to prevent cryptococcal meningitis from occurring or encourage early health-seeking behaviours. As discussed, only one of the participants who knew they were living with HIV had mentioned that they knew meningitis could be a potentially serious complication of untreated infection. No participants mentioned having received any specific information or education about meningitis whilst accessing HIV care. In addition, we found that only one of the participants had attended their usual HIV clinic whilst seeking care for their headache. In this instance a 23-year-old female participant in Kampala was told that her symptoms were likely due to taking her ART at night and was advised to change to morning dosing. It was only after the symptoms became more severe and the participant was brought back to the clinic in a coma that she was transferred to hospital.

Several participants had very recently initiated ART and developed their headache within the first few months of starting treatment. Despite this they often presented to other healthcare facilities, rather than their HIV clinics, when they developed symptoms and they did not report having been told that cryptococcal meningitis and other infections such as tuberculosis can sometimes only develop shortly after treatment is initiated. There were also two instances where participants were diagnosed with HIV whilst suffering from a headache but were started on ART rather than being investigated and managed for cryptococcal meningitis.

Finally, the Zimbabwean participants who were recruited in Gaborone were, at the time of the study, not able to access free ART in Botswana and so either had to pay in Botswana or travel to Zimbabwe to access it for free. One 29-year-old female participant explained that due to stock outs in Zimbabwe she had not been able to access her usual ART regimen and had been put back on a regimen which she had previously stopped due to side-effects. When the same side-effects occurred, she stopped the regimen and eventually developed cryptococcal meningitis.

## 4 Discussion

In this qualitative methods study of participants in a clinical trial for HIV-associated cryptococcal meningitis we found that pathways to care were prolonged for several reasons. First, headaches are a common complaint, typically without severe consequences, and are often attributed to environmental factors such as hydration and psychological wellbeing. Where headaches are caused by biomedical aetiologies, including infections, the differential diagnosis is broad and there are multiple therapeutic options that can be easily accessed. Second, people living with HIV are not well informed about the possibility for headaches to signify a serious underlying pathology in the context of AHD and so meningitis is very rarely suspected. Third, healthcare workers who do not specialise in HIV do not always suspect meningitis as the cause of a headache and this is much harder if they are unaware of their patient's HIV status. Finally, HIV clinicians do not always inform patients about meningitis, particularly around the time of ART initiation, and can sometimes cause harm by prescribing ART to patients with symptoms of meningitis.

There is an urgent need to recognise cryptococcal meningitis as early as possible. As we have discussed the absolute mortality risk in the AMBITION-cm trial was more than twice as high in those who were diagnosed whilst suffering from confusion or reduced consciousness. The ubiquity of headaches and their broad differential diagnosis can lead to cognitive biases among healthcare workers which were observed within this study. We observed multiple alternatives bias whereby the number of possible aetiologies considered by healthcare workers can be overwhelming and is subsequently simplified to a smaller, manageable subset with which they are familiar (Redelmeier & Shafir, 1995). This has previously been described as a common challenge when managing individuals with headaches and can lead to a lack of consideration of other, potentially more serious pathologies (Gottschalk, 2019).

Within our data we observed that many of the participant's pathways to care were quite similar, in that the standard approach seemed to be to advise hydration and provide simple analgesia, then consider common pathologies such as flu, malaria or raised blood pressure, then think of another, one by one, almost in a syndromic, trial-and-error manner. This is likely a tried and tested approach which works for the majority of individuals but when less commonly encountered pathologies occur, as is the case in this study, it can lead to vertical line failure whereby there is a lack of lateral thinking or a consideration of ‘what else could this be?’ (Croskerry, 2002). Finally, we assume that as healthcare workers are likely to see many individuals with headaches, in the majority of cases the symptom is self-limiting or responsive to commonly prescribed treatments. This can lead to posterior probability error whereby if the previous approach has worked many times before then it will likely work in this scenario too (Hansen et al., 2021). These heuristics are common in HIV medicine and are certainly not limited to our geographical context, having been described in encounters elsewhere (Deming et al., 2019).

A critical step that propelled these pathways to care and the ultimate diagnosis of cryptococcal meningitis was the recognition of the individual's HIV status and that they were likely to be living with AHD. Those who had previously been diagnosed with HIV did have a lower median number of previous healthcare interactions prior to being admitted to hospital. This recognition of AHD can be achieved with regular HIV testing but also requires an acknowledgement that people living with HIV can move in both directions along the care cascade and therefore those who are or who have previously been receiving ART can develop AHD. Data from Botswana show that this is an increasingly large proportion of people with AHD and we anticipate it will continue to grow over time (Lawrence et al., 2021a). Recognition of AHD is more difficult in situations of non-disclosure of HIV status, a phenomenon we observed within this study. There is extensive research that has explored the concept of non-disclosure and demonstrated an association with negative outcomes (Akilimali et al., 2017; Arrivé et al., 2012). Within this study we observed evidence of non-disclosure to family and friends and also to healthcare workers. Reasons given for non-disclosure to healthcare workers have included concerns around confidentiality and stigma (Greeff et al., 2008) as well as not feeling that disclosure was necessary in a particular context (Agne et al., 2000). In addition, some of our participants showed evidence of having not yet accepted their HIV status, having gone back to test on multiple occasions, sometimes without informing healthcare workers that they had tested positive in the past. Again, this is a well described phenomenon (Horter et al., 2017; Nam et al., 2008; Wringe et al., 2009).

We have identified a number of key foci for educational interventions that can help facilitate the prevention, identification, and management of cryptococcal meningitis. First, patients and their friends and family need to know about the potentially severe complications of untreated HIV disease so that they can be aware that a headache may not be so ‘simple’ for them and that certain symptoms that develop shortly after ART should prompt rapid presentation to a HIV clinic or hospital. The information that they receive, how it is communicated, and using which methods, needs to be developed by communities of people living with HIV in order to be effective and there are several examples of best practice in this area (AfroCAB, 2021; Differentiated Service Delivery, 2022). Second, healthcare workers who are not HIV specialists need to know how to recognise AHD, both clinically but also by using rapid diagnostic tests which should be made available to them. Third, healthcare workers at HIV clinics needs to ensure that their clients are aware of cryptococcal meningitis and that ART prescribing is done safely, in the absence of any symptoms that could suggest meningitis, and with adequate safety-netting should those symptoms develop.

When the diagnosis of AHD is made or known a whole new differential diagnosis gains prominence along with a new syndromic approach to diagnosis and management. Differentiated service delivery models for HIV care have gained traction since 2015 but have typically focused on innovative ways to deliver care to stable outpatients (Grimsrud et al., 2016). Differentiated service delivery models for AHD specifically have only started to gain prominence in recent years and thus far primarily focus on the availability of a package of rapid diagnostic tests for CD4 count, cryptococcal disease and tuberculosis, coupled with therapeutics to prevent and treat these infections (Differentiated Service Delivery, 2019). These programmes adopt a hub-and-spoke model with local centres implementing the majority of the package and then referring to inpatient units when acute care is required. Significant progress has been made by these programmes in last few years, particularly with cryptococcal disease. A partnership between Clinton Health Access Initiative and Unitaid has provided diagnostics and antifungal medications, including flucytosine and liposomal amphotericin, to countries with a high incidence of cryptococcal meningitis (Unitaid, 2021), and early observational data are promising (Clinton Health Access Initiative Unitaid, 2022). This programme underscores the need to provide access to diagnostics and therapeutics for AHD that are freely available for all. For example, we described the particular vulnerability of Zimbabwean participants in Botswana who during the earlier phase of the trial were not eligible for free HIV care. Since 2019 this policy has been reversed and ART is now freely available to non-citizens in Botswana, a move that was lauded by key stakeholders in the HIV response (UNAIDS, 2019).

This acknowledgement of the differentiated service needs of people living with AHD is extremely welcome and this research study can inform the future development of such approaches. First, we observed a clear centralisation of knowledge and expertise around cryptococcal meningitis, insomuch that once the diagnosis was considered it was often clear that participants needed to be urgently referred to central locations. However, participants were regularly moved from one hospital to another to initiate treatment, putting considerable time and distance between themselves and their first dose of antifungal medication. In addition to skilling up healthcare workers around AHD at all facilities there is a need to decentralise care so that more hospitals are equipped with the skills and resources to offer rapid, high-quality care. This would work in synergy with the hub and spoke model. Second, differentiated service delivery models for AHD have thus far been almost entirely biomedical in nature, providing the essential diagnostics and therapeutics but overlooking the sociological context of AHD, particularly among individuals who have known their HIV diagnosis for some time. There is an urgent need to develop and integrate evidence based social and behavioural interventions into these programmes as a standard. When combined with effective diagnostics and therapeutics these can be life-saving interventions that prevent the persistence or recurrence of AHD and ultimately reduce mortality.

There are limitations to this study. The trial participants were very unwell with a life-threatening neurological infection, even those who were decision-orientated at baseline, so it is likely there was some recall bias as a result. This is particularly true for some trial participants who had their pathways to care narrated back to them by other people who had escorted them as they simply had no memory. In addition, we observed inconsistencies between what was recorded in trial documents by the AMBITION-cm research team and collected through the in-depth interviews, particularly in terms of HIV and ART status, suggesting that some participants knew their HIV status and/or had been prescribed ART before. Within some interviews we also observed that participants were not always comfortable talking to us about their previous or current ART adherence. We therefore conclude that the findings of this analysis are also subject to the same response bias. Finally, all data collected in Setswana or Luganda were translated to English so the nuance of some testimony will have been lost, however each interview was discussed within the core team to try and reduce this.

## 5 Conclusion

We found that pathways to care with cryptococcal meningitis were prolonged because headaches were often disregarded as an everyday occurrence and had a broad differential diagnosis of predominantly benign aetiologies. There was a lack of awareness of the disease among participants and healthcare workers and it was typically only when a diagnosis of HIV was made or disclosed that the diagnostic pathway accelerated and resulted in hospital admission. We have outlined key recommendations to prevent, diagnose and manage cryptococcal meningitis and argued for the integration of social and behavioural interventions into differentiated service delivery models for advanced HIV disease.

## Funding

This study was funded by the National Institute for Health Research (NIHR) through a Global Health Research Professorship to JNJ (RP-2017-08-ST2-012) using UK aid from the UK Government to support global health research. The AMBITION-cm trial, including salary support for DSL, DM, TSH and JNJ was supported by a grant through the European Developing Countries Clinical Trials Partnership (EDCTP), the Swedish International Development Cooperation Agency (SIDA) (TRIA2015-1092), and the Wellcome Trust/Medical Research Council(UK)/UKAID Joint Global Health Trials (MR/P006922/1). The views expressed in this publication are those of the authors and not necessarily those of the funders.

## Ethics approval

The study was approved by the Human Resource Development Council, Gaborone (HPDME: 13/18/1); Makerere School of Health Sciences Institutional Review Board, Kampala (REF: 2019-061), Uganda National Council for Science and Technology (REF: SS386ES) and the London School of Hygiene and Tropical Medicine (REF: 17957).

## Consent to participate

All participants signed a written consent form which was approved by the relevant research ethics committees.

## Consent for publication

All participants provided written consent for publication, including the use of anonymised quotes.

## Availability of data and material

Anonymised data are available by reasonable request to the corresponding author.

## Authors contributions

David S Lawrence: Conceptualization, Methodology, Formal Analysis, Investigation, Resources, Data Curation, Writing – Original Draft, Project Administration. Agnes Ssali: Conceptualization, Methodology, Formal Analysis, Writing – Review & Editing, Project Administration. Neo Moshashane: Formal Analysis, Investigation, Data Curation, Writing – Review & Editing, Project Administration. Georgina Nabaggala: Formal Analysis, Investigation, Data Curation, Writing – Review & Editing, Project Administration. Lebogang Maphane: Data Curation, Project Administration. Thomas S Harrison: Methodology, Writing – Review & Editing, Supervision. David Meya: Methodology, Resources, Writing – Review & Editing, Supervision. Joseph N Jarvis: Conceptualization, Methodology, Resources, Writing – Review & Editing, Supervision, Funding acquisition. Janet Seeley: Conceptualization, Methodology, Formal Analysis, Resources, Writing – Original Draft, Supervision.

## Declaration of competing interest

The authors declare that they have no known competing financial interests or personal relationships that could have appeared to influence the work reported in this paper.
